# A Normotensive Case of Pheochromocytoma With Unusual Presentation of Abdominal Pain

**DOI:** 10.7759/cureus.47063

**Published:** 2023-10-15

**Authors:** Amina Abid, Ahmed Imran Siddiqi, Waqas Shafiq, Hira Irfan

**Affiliations:** 1 Internal Medicine, Shaukat Khanum Memorial Cancer Hospital and Research Centre, Lahore, PAK; 2 Endocrinology and Diabetes, Shaukat Khanum Memorial Cancer Hospital and Research Centre, Lahore, PAK

**Keywords:** parathyroid adenoma, normotensive, mtc, men 2a, pheochromocytoma

## Abstract

Multiple endocrine neoplasia (MEN) is an inherited, autosomal dominant condition characterized by primary parathyroid hyperplasia, medullary thyroid neoplasm, and pheochromocytoma. It most commonly presents with medullary thyroid cancer and less frequently with other complaints. Pheochromocytoma can also manifest through gastrointestinal complaints such as abdominal pain, nausea, and constipation. We present a normotensive case of pheochromocytoma, initially featuring abdominal pain and vomiting, which was later found to be associated with neck swelling and medullary thyroid cancer. The patient underwent an adrenalectomy and has continued to visit our endocrinology clinic for ongoing monitoring and treatment of iatrogenic hypoparathyroidism and hypothyroidism. A brief review is also provided.

## Introduction

Multiple endocrine neoplasia (MEN) is an autosomal dominant, inherited condition characterized by primary parathyroid hyperplasia, medullary thyroid neoplasm, and pheochromocytoma. In the MEN 2A subtype, pheochromocytoma is the initial manifestation in only 15% of cases, and 25% present with concomitant pheochromocytoma and medullary thyroid cancer (MTC) [1]. According to one study, 95% of patients diagnosed with MEN 2A develop MTC, 50% develop pheochromocytoma, and approximately 20-30% develop hyperparathyroidism [2]. MEN 2A arises due to a germline mutation in the RET protooncogene. A total of 50% of patients harboring this mutation develop the disease by the age of 50 years and 70% by the age of 70 years [3]. Nevertheless, MEN syndromes impact individuals of all ages, and both sexes are equally affected [4]. Screening first-degree relatives proves beneficial for early diagnosis and treatment. The prognosis typically hinges on the stage of the medullary thyroid neoplasm at diagnosis. It is a rare familial cancer with an estimated prevalence of 1 in 35,000. Pheochromocytoma is a rare neuroendocrine tumor, originating from the adrenal medulla or extra-adrenal paraganglion chromaffin tissue, and is characterized by catecholamine secretion [5]. While its estimated incidence ranges from 0.005% to 0.1% in the general population and 0.1% to 0.2% among the adult hypertensive population, these figures account for only 50% of individuals with pheochromocytoma. This is because approximately half of the affected individuals either exhibit normotension or experience paroxysmal hypertension [5]. Pheochromocytomas occur most frequently in individuals aged 40-50 years [6], with a slight predilection in females (55.2%) than men (44.8%) [7]. Owing to their rarity and variable manifestations, these tumors pose significant diagnostic challenges. Consequently, many are incidentally discovered during radiological examinations, particularly of the abdomen, as adrenal incidentalomas, or identified posthumously during autopsies [8]. According to Gifford RW et al., abdominal pain presents in 14% of pheochromocytoma cases [9]. Nonetheless, nausea is often suggested as the prevailing gastrointestinal symptom [6], while constipation is also recorded as a chronic issue in 13% of patients [10].

## Case presentation

**Figure 1:**
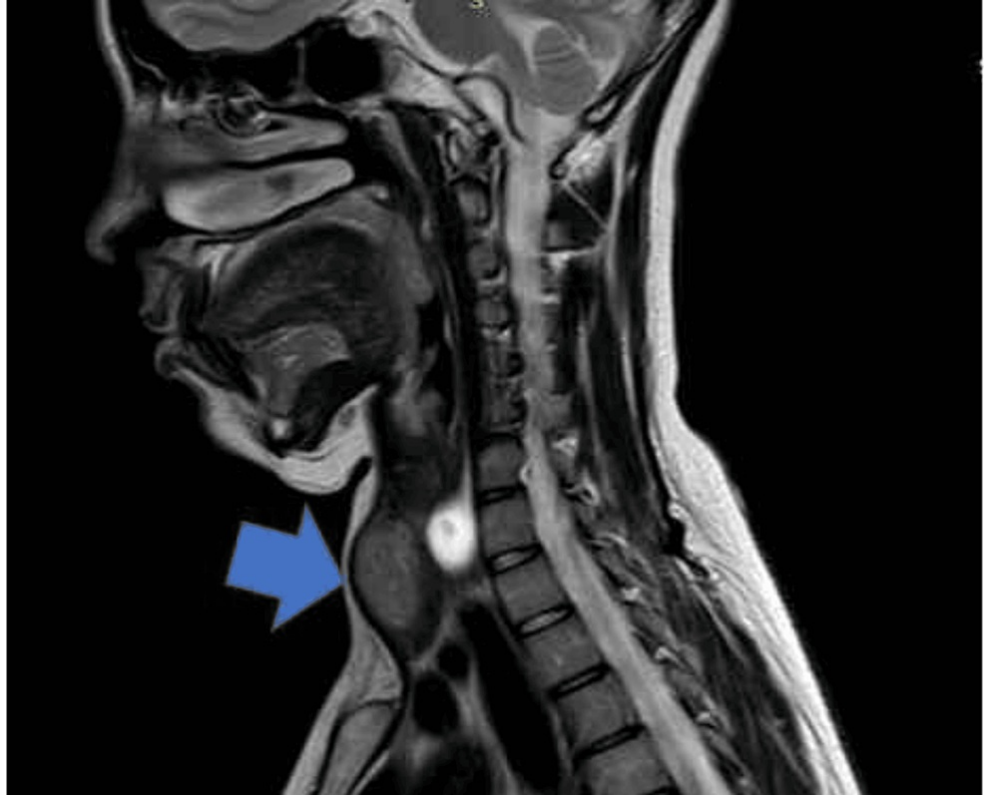
MRI neck without contrast showing thyroid mass.

We report a case of a 25-year-old female, married, with two kids, non-pregnant, non-lactating, presented with abdominal pain for two years. The pain was localized, sharp, severe, episodic, associated with vomiting when severe, and not relieved by antacids or analgesics. Her initial workup and treatment for common causes of abdominal pain did not find a definite cause of her pain. She also complained of palpations, headaches, sweating, and neck swelling for seven years with no recent increase in size. Her mother also suffered from neck swelling but was never investigated for it. There was no history of malignant or endocrine disease in the family. She had a 5 x 5 cm midline neck mass with no palpable lymph nodes (Figure 1), was completely normotensive, and had never had problems during her pregnancies or in the postpartum period. 

**Figure 2:**
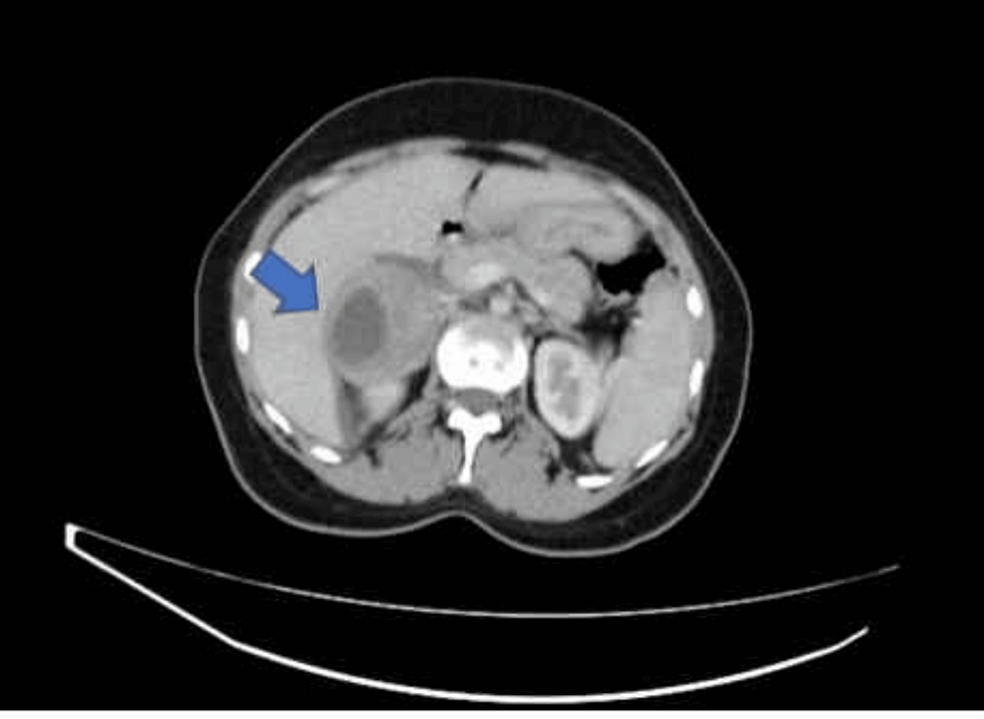
CT abdomen and pelvis with contrast showing right-sided adrenal mass.

A CT scan of the abdomen and pelvis revealed a right suprarenal lesion, raising concerns about a potential malignant adrenal lesion or pheochromocytoma, given its size, heterogeneity, and internal necrosis (Figure 2). Subsequent investigations were conducted, revealing significantly elevated levels of metanephrine, normetanephrine, calcitonin, and carcinoembryonic antigen (CEA). Additionally, serum calcium was at the upper limit of normal, accompanied by elevated parathyroid hormone (PTH) levels. An ultrasound of the neck identified a nodule on the right side.

**Figure 3:**
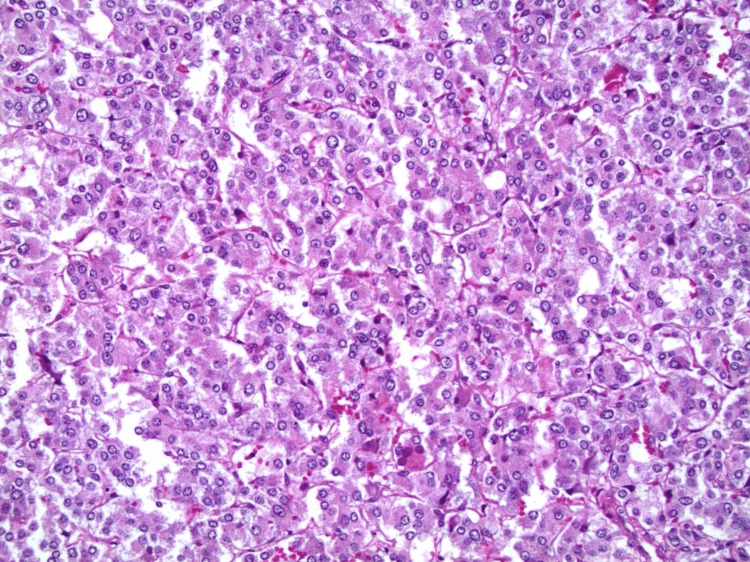
Histopathology of pheochromocytoma.

She was later planned for right adrenalectomy after adequate alpha and beta blockage, followed by thyroid surgery. Histopathology confirmed a Pheochromocytoma of the Adrenal gland Scaled Score (PASS) of 0-1, revealing a nesting (Zellballen) pattern upon microscopy. This pattern comprised well-defined clusters of tumor cells, characterized by eosinophilic cytoplasm, and was separated by a fibrovascular stroma (Figure 3).

Post-operative metanephrine and normetanephrine levels were normal, with normal PTH (Table 1) and low vitamin D levels. Subsequently, Vitamin D was replaced, and a total thyroidectomy and an excision of the parathyroid nodule were conducted. The histopathology validated the presence of medullary thyroid cancer and a parathyroid adenoma.

**Table 1:** Biochemical results. PTH: Parathyroid hormone; ND: Not detected.

Normal values	Pre-operatively	Post-operatively
Metanephrine (Less than 90)	>650 pg/mL	16.79 pg/mL
Normetanephrine (Less than 190)	>900 pg/mL	112.2 pg/mL
Calcitonin (ND: 5.0)	5868 pg/mL	<2 pg/mL
Calcium (8.6-10)	10.61 mg/dL	9.77 mg/dL
PTH (18.5-88)	95.1 pg/mL	49 pg/mL

She continued to visit our endocrinology clinic with ongoing monitoring and treatment of her iatrogenic hypoparathyroidism and hypothyroidism. She is also being monitored for the recurrence of her medullary thyroid cancer and pheochromocytoma. 

Learning points 

Abdominal pain is a common presenting symptom for many clinical conditions. One should consider serious diagnoses like pheochromocytoma while investigating such patients after ruling out common causes of abdominal pain. We recommend screening young patients with a confirmed diagnosis of pheochromocytoma for MEN utilizing tests such as neck ultrasound, thyroid profile, and assessments of Parathyroid Hormone (PTH) and calcium levels. This is pivotal to preclude overlooking the triad of malignancy in associated organs, such as medullary thyroid cancer or parathyroid adenoma.

## Discussion

We report a case of unilateral, benign pheochromocytoma with an unusual presentation of abdominal pain instead of more common presentations of palpitations, headaches, and sweating. Abdominal pain is a quite common manifestation in patients presenting to the ED. Her initial workup and treatment for common causes of abdominal pain did not find a definite cause of her abdominal pain. Although pheochromocytoma usually presents with episodic headache, sweating, and tachycardia, a few cases present with acute abdominal pain. According to the literature, 14% of patients with pheochromocytoma present with abdominal pain [9]. The pain can primarily arise from the mass effect on internal viscera, hemorrhagic necrosis, or rupture of the tumor [11]. One thing to be noted is that our patient had never had any problems during her pregnancies or in the postpartum periods, probably because she did not have pheochromocytoma at the time of pregnancy because several mechanisms can trigger clinically overt pheochromocytoma in pregnancy. These include increases in intra-abdominal pressure, fetal movement, uterine contraction, delivery process, abdominal surgical intervention, and even general anesthesia [12]. She had no known family history of cancer or endocrine disease. Although pheochromocytomas are often hereditary, many patients with hereditary forms of pheochromocytomas will not have a family history of the disease. However, people with hereditary forms of pheochromocytomas may be at risk of developing other tumors that may be a part of a known hereditary syndrome like MEN [13], like in our case, where our patient was later diagnosed with medullary thyroid cancer. It should also be highlighted here that our patient was normotensive both at presentation and during her entire course of therapy. Although hypertension, sustained or paroxysmal, is usually a cardinal feature of pheochromocytoma, normotensive presentation is unusual. Roy M et al. present a case of normotensive pheochromocytoma with a management dilemma [14]. According to them, hypertension may be absent in pheochromocytomas despite excess norepinephrine secretion [14]. So, in the absence of hypertension, using a combination of alpha and beta-blocker therapy may cause a precipitous fall in blood pressure; therefore, these agents should be used cautiously in such patients [14]. 

Calcitonin was checked for the first time because of neck mass; however, interestingly, neck mass was there for seven years. Although pheochromocytoma is usually the first manifestation of MEN syndrome, we must be careful about the development of medullary thyroid carcinoma, which is an aggressive tumor with rapid metastasis, and the MTC component of the disease is often what determines the long-term course of MEN 2A. On one end of the scale, this may be treated with prophylactic thyroid surgery in a young child before MTC even manifests. On the other, a patient may experience their first diagnosis of MTC in adulthood, which may have already spread far. Patients with all the MTC removed at the first surgery or who get an early preventive thyroidectomy have excellent prognoses [15]. In 10-15% of cases, MTC is only discovered after thyroid surgery. This delay in diagnosis may have adverse effects, such as failing to detect an underlying pheochromocytoma or hyperparathyroidism in type 2 MEN or performing surgery with insufficient precision [16]. 

Literature available on pheochromocytoma mostly emphasizes early diagnosis, management, and family screening in patients with pheochromocytoma and MEN syndromes. However, not many studies highlight the abdominal presentation of pheochromocytoma and the importance of considering a serious diagnosis like pheochromocytoma while investigating abdominal pain because misdiagnosis leads to high morbidity and mortality. A case report by Sweeney AT et al. highlights the abdominal features of pheochromocytoma at presentation. According to them, pheochromocytoma can present with GI symptoms such as nausea, vomiting, and abdominal pain. In a wider series, constipation was recorded in 5-13% of the cases. Pheochromocytoma and a megacolon may co-occur in MEN 2A and MEN 2B. Congenital a-ganglionic colon, often known as Hirschsprung's disease, has been linked to both MEN 2A and 2B syndromes [17]. Hence, careful history taking, critical interpretation of each diagnostic test, alternative review of functional examinations, and anatomic imaging are essential for a correct diagnosis. 

Regular follow-up is crucial to ensure early detection of any pheochromocytoma development since the elevated blood pressure induced by these tumors can lead to significant side effects, including stroke and heart attack. However, these abnormalities can be resolved early with routine supervision in a specialized clinic, ensuring they are managed early before becoming problematic. Moreover, if a patient is diagnosed with pheochromocytoma or MTC, it is imperative to screen associated glands to potentially diagnose MEN, particularly in younger patients.

## Conclusions

Prompt diagnosis and treatment of MEN 2A, which can be facilitated by early screening of family members of confirmed patients, play a pivotal role in reducing morbidity and mortality. We did recommend family screening for our patient, but the patient's family denied screening. Moreover, if MEN 2A is diagnosed early and both surgeries, i.e., adrenalectomy and thyroidectomy, are planned, adrenalectomy should be performed first to avoid catecholamine release intraoperatively. Physicians should pay more attention to the clinical manifestations of MEN and thorough clinical examination to avoid unnecessary delays in diagnosis and management. Awareness should be provided in the general population regarding its clinical manifestations and the importance of family screening. In instances where a patient is diagnosed with pheochromocytoma or MTC, screening for additional MEN diagnoses should always be performed, particularly in younger patients.
